# Apixaban outcomes in atrial fibrillation patients with a single-dose reduction criterion: ASPIRE 1-year results

**DOI:** 10.1093/ehjcvp/pvaf018

**Published:** 2025-03-20

**Authors:** So-Ryoung Lee, JungMin Choi, Soonil Kwon, Hyo-Jeong Ahn, Kyung-Yeon Lee, Jong-Il Choi, Sung Ho Lee, Jung Ho Heo, Il-Young Oh, Young Keun On, Hee Tae Yu, Kwang-No Lee, Nam-Ho Kim, Hyung Wook Park, Ki Hong Lee, Seung Yong Shin, Hyoung-Seob Park, Seongwook Han, Seil Oh, Gregory Y H Lip, Jong-Sung Park, Eue-Keun Choi

**Affiliations:** Department of Internal Medicine, Seoul National University Hospital, 101, Daehak-ro, Jongno-gu, Seoul 03080, Republic of Korea; Department of Internal Medicine, Seoul National University College of Medicine, 103, Daehak-ro, Jongno-gu, Seoul 03080, Republic of Korea; Department of Internal Medicine, Seoul National University Hospital, 101, Daehak-ro, Jongno-gu, Seoul 03080, Republic of Korea; Department of Internal Medicine, Seoul National University Hospital, 101, Daehak-ro, Jongno-gu, Seoul 03080, Republic of Korea; Department of Internal Medicine, Seoul National University Hospital, 101, Daehak-ro, Jongno-gu, Seoul 03080, Republic of Korea; Department of Internal Medicine, Seoul National University Hospital, 101, Daehak-ro, Jongno-gu, Seoul 03080, Republic of Korea; Department of Internal Medicine, Division of Cardiology, Korea University College of Medicine and Korea University Anam Hospital, Seoul 02841, Republic of Korea; Division of Cardiology, Department of Internal Medicine, Kangbuk Samsung Hospital, Sungkyunkwan University School of Medicine, Seoul 03181, Republic of Korea; Department of Internal Medicine, Kosin University Gospel Hospital, Busan 49267, Republic of Korea; Department of Internal Medicine, Seoul National University Bundang Hospital, Gyeonggi 13620, Republic of Korea; Department of Cardiology, Heart Vascular Stroke Institute, Samsung Medical Center, Sungkyunkwan University School of Medicine, Seoul 06351, Republic of Korea; Department of Internal Medicine, Severance Cardiovascular Hospital, Yonsei University College of Medicine, Seoul 03722, Republic of Korea; Department of Cardiology, Ajou University School of Medicine, Suwon 16499, Republic of Korea; Department of Internal Medicine, Wonkwang University Hospital, Iksan 54538, Republic of Korea; Department of Internal Medicine, Chonnam National University Hospital, Gwangju 61469, Republic of Korea; Department of Cardiovascular medicine, Chonnam National University Medical School, Gwangju 61469, Korea; Department of Internal Medicine, Chonnam National University Hospital, Gwangju 61469, Republic of Korea; Department of Cardiovascular medicine, Chonnam National University Medical School, Gwangju 61469, Korea; Cardiovascular & Arrhythmia Center, Chung-Ang University Hospital, Seoul 06973, Republic of Korea; Division of Cardiology, Department of Internal Medicine, Keimyung University Dongsan Hospital, Daegu 41931, Republic of Korea; Gangsim Heart Clinic, Daegu 41942, Republic of Korea; Department of Internal Medicine, Seoul National University Hospital, 101, Daehak-ro, Jongno-gu, Seoul 03080, Republic of Korea; Department of Internal Medicine, Seoul National University College of Medicine, 103, Daehak-ro, Jongno-gu, Seoul 03080, Republic of Korea; Department of Internal Medicine, Seoul National University College of Medicine, 103, Daehak-ro, Jongno-gu, Seoul 03080, Republic of Korea; Liverpool Center for Cardiovascular Science at University of Liverpool, Liverpool John Moores University and Liverpool Chest and Heart Hospital, Liverpool L7 8TX, UK; Department of Clinical Medicine, Aalborg University, Aalborg 9220, Denmark; Department of Cardiology, Dong-A University Hospital, 26, Daesingongwon-ro, Seo-gu, Busan 49201, Republic of Korea; Department of Internal Medicine, Seoul National University Hospital, 101, Daehak-ro, Jongno-gu, Seoul 03080, Republic of Korea; Department of Internal Medicine, Seoul National University College of Medicine, 103, Daehak-ro, Jongno-gu, Seoul 03080, Republic of Korea

**Keywords:** Atrial fibrillation, Apixaban, On-label standard dose, Off-label reduced dose

## Abstract

**Aims:**

This study, using a prospective cohort, evaluated the effectiveness and safety of off-label reduced-dose apixaban vs. the on-label dose in atrial fibrillation (AF) patients meeting a single-dose reduction criterion.

**Methods and results:**

The efficAcy and Safety of aPixaban In REal-world practice in Korean frail patients with AF (ASPIRE) study is a multicentre, prospective observational cohort involving AF patients who met a single-dose reduction criterion of apixaban. Patients were divided into two groups: an on-label standard dose (5 mg twice daily) and an off-label reduced dose (2.5 mg twice daily). The primary effectiveness outcome was stroke/systemic embolism (SSE), and the primary safety outcome was major bleeding. Of 1944 patients (mean age 74.3 ± 7.9 years, 56% women), 997 (51%) were receiving off-label reduced-dose apixaban. The off-label reduced-dose group was older, had more comorbidities, higher concomitant antiplatelet use, and higher CHA_2_DS_2_–VASc and HAS-BLED scores. During follow-up (1.0 ± 0.2 year), crude incidence rates were 0.9 vs. 0.7 per 100 person-years for SSE and 0.5 vs. 1.0 for major bleeding in the on-label vs. off-label groups. After inverse probability of treatment weighting, the off-label reduced-dose group showed no significant differences in the risk of SSE [hazard ratio (HR) 0.67, 95% confidence interval (CI) 0.28–1.59, *P* = 0.370] and major bleeding (HR 1.38, 95% CI 0.44–4.35, *P* = 0.578) compared with the on-label standard dose group.

**Conclusion:**

In Korean patients with AF meeting a single-dose reduction criterion of apixaban, off-label reduced-dose apixaban showed no significant differences in SSE and major bleeding compared with the on-label standard dose. These findings suggest that individualized anticoagulation strategies, such as reduced-dose apixaban, may be beneficial for patients with a high risk of bleeding.

## Graphical Abstract

**Graphical Abstract:**
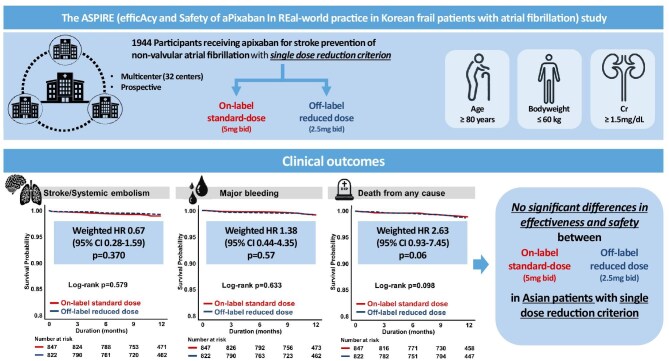
Clinical outcomes of apixaban in atrial fibrillation patients with a single-dose reduction criterion: 1-year outcome of the ASPIRE study. In a prospective cohort study of 1944 Asian atrial fibrillation patients meeting a single-dose reduction criterion for apixaban, off-label reduced-dose apixaban showed no significant differences in the risk of stroke/systemic embolism, major bleeding, or all-cause mortality compared with the on-label standard dose. Abbreviation: CI, confidence interval; HR, hazard ratio.

## Introduction

Oral anticoagulants (OACs), particularly direct oral anticoagulants (DOACs), are recommended for stroke prevention in atrial fibrillation (AF) patients.^1–3^ The primary recommendation for reducing the dosage of DOACs is based on the officially published and approved criteria for dose reduction.^4^ In the pivotal ARISTOTLE trial, apixaban at the standard dose (5 mg twice daily) was compared with warfarin in patients with AF, while a reduced dose of apixaban (2.5 mg twice daily) was reserved for those meeting at least two of the following criteria: age ≥80 years, body weight ≤60 kg, or serum creatinine ≥1.5 mg/dL.^5^ In the ARISTOTLE trial, a total of 428 patients received apixaban 2.5 mg or placebo (4.7% in the apixaban group) according to the above dose reduction criteria. Despite guidelines advising against reducing DOAC doses solely due to bleeding concerns, off-label underdosing is common in clinical practice.^6–8^ Several retrospective studies have suggested that off-label reduced-dose DOACs may increase the risk of stroke and/or mortality without reducing bleeding risk.^6,9,10^ Notably, for apixaban, such off-label underdosing has been associated with up to a five-fold increase in stroke risk compared with the recommended dose.^9^

In Korean populations, off-label reduced-dose apixaban has been associated with increased stroke risk compared with the on-label standard dose.^8,11^ Nevertheless, among patients meeting only one of the aforementioned criteria, there was no significant difference in the risk of ischaemic stroke and major bleeding between those receiving the on-label standard dose and those receiving off-label reduced-dose apixaban.^11^ However, there are limited prospective data on the clinical impact of off-label reduced-dose apixaban in patients at high risk for bleeding who meet the single-dose reduction criterion.

This study assessed the effectiveness and safety of off-label reduced-dose apixaban compared with the on-label dose apixaban in AF patients, meeting the single-dose reduction criterion for apixaban in a prospective, multicentre, non-interventional cohort.

## Methods

A more detailed description of the methods is presented in the Supplementary material online, Supplemental Methods section. The ASPIRE (efficAcy and Safety of aPixaban In REal-world practice in Korean frail patients with AF) study was a prospective, multicentre, non-interventional observational investigation encompassing all geographic areas within South Korea across 32 participating centres. The study protocol was approved by each centre’s ethics committee and adhered to the principles of the Declaration of Helsinki (H-2108-110-1245). It was registered on ClinicalTrials.gov (NCT05773222), and informed consent was obtained from all participants.

### Study population and design

Participants 19 years or older with non-valvular AF, who were prescribed apixaban and met the single-dose reduction criterion, were screened. The criteria for apixaban dose reduction included: (i) age ≥80 years, (ii) body weight ≤60 kg, and (iii) serum creatinine level ≥1.5 mg/dL.^5^ Exclusion criteria comprised: (i) vulnerability (as defined by Korean Good Clinical Practice) or disagreement with the study, (ii) patients who experienced clinical events, as outlined in the primary and secondary outcomes of the study, prior to enrolment while on apixaban, and (iii) meeting two or more dose reduction criteria for apixaban. The choice of apixaban dosage—5 mg twice daily (standard dose) or 2.5 mg twice daily (reduced dose)—was at the discretion of the treating physicians.

### Covariates

Demographic data, anthropometric measurements, blood pressure, and heart rate were recorded. Baseline variables included comorbidities such as hypertension, diabetes mellitus, heart failure, history of stroke/transient ischaemic attack (TIA), bleeding, chronic kidney disease (CKD), liver disease, and malignancy. Laboratory findings encompassed complete blood count, coagulation parameters, and renal function tests. CHA_2_DS_2_–VASc and HAS-BLED scores were calculated using participants’ comorbidities and laboratory data.^12^ AF diagnosis included type, European Heart Rhythm Association (EHRA) symptom classification, and rhythm control status. Baseline pharmacological treatment data and anaemia status were also documented. All available data for each analysis were used without excluding entire cases.

### Follow-up and outcomes

The study was prospectively followed up to 12 months after enrolment, with recommended data collection every 3 months. The primary effectiveness outcome was the first stroke/systemic embolism (SSE) during follow-up. Secondary effectiveness outcomes included TIA, myocardial infarction, cardiovascular death, all-cause death, and a composite of thrombo-embolic events (SSE, TIA, and myocardial infarction). The primary safety outcome was the first major bleeding (the International Society on Thrombosis and Haemostasis criteria), with secondary safety outcomes including a composite of major bleeding and clinically relevant non-major bleeding (CRNMB), and a composite of major bleeding, CRNMB, and minor bleeding events.^13^ Apixaban dosing status, dose changes, and 12-month laboratory tests were monitored during follow-up.

### Statistical methods

Regarding baseline characteristics, continuous variables are expressed as mean ± standard deviation, while categorical variables are presented as numbers and percentages. Group comparisons were conducted using various statistical tests including the Mann–Whitney *U*, χ^2^, analysis of variance, and Fisher's exact tests.

Incidence rates (IRs) were calculated as the number of events per 100 person-years (PY). Survival analysis using the Kaplan–Meier method (log-rank test) and Cox proportional-hazards regression assessed the risks of primary and secondary outcomes between the off-label low-dose and on-label standard-dose apixaban groups (reference). Hazard ratios (HRs) with 95% confidence intervals (CIs) were calculated with stepwise adjustments: unadjusted, adjusted for age, sex, comorbidities, concomitant antiplatelet use, and anaemia. Furthermore, to compare the clinical outcomes between the two groups after balancing the differences of baseline characteristics of the two groups, we performed an inverse probability of treatment weighting (IPTW) analysis.^14^ Propensity scores were generated using logistic regression, with matching variables including demographics, comorbidities, and medications.^14,15^ Standardized mean differences ≤0.1 indicated well-balanced groups.^16^ Post-matching survival analysis used log-rank tests and Cox proportional-hazards regression models, accounting for calculated weights.^15^

Statistical significance was defined as *P* < 0.05. All statistical analyses were conducted utilizing SPSS version 25 (IBM Corp., Armonk, NY, USA).

### Subgroup analysis

Subgroup analyses for SSE, death from any cause, and major bleeding compared on-label standard dose to off-label reduced-dose apixaban across age categories, body weight, serum creatinine levels, CHA_2_DS_2_–VASc score, HAS-BLED score, and CKD Epidemiology Collaboration equation estimated glomerular filtration rate (EPI eGFR). These analyses used a multivariable Cox proportional-hazards regression model with adjustments.

## Results

**Figure 1:**
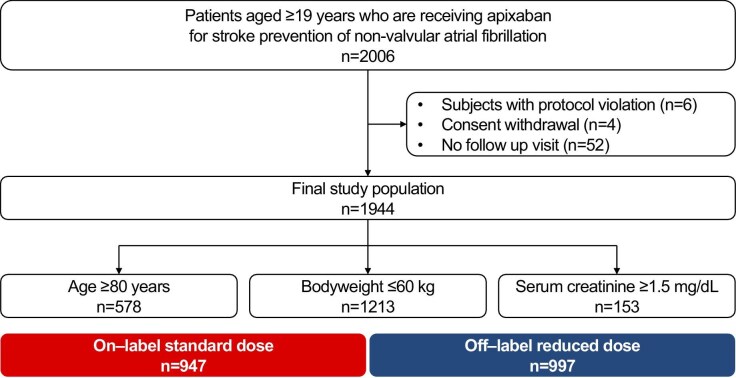
Study flow. The overall enrolment flow is presented.

*Figure 1* depicts the study flow. From March 2020 to September 2022, a total of 2006 patients were enrolled. After exclusions due to protocol violations, consent withdrawal, and loss to follow-up, 1944 patients were included in the analysis. Enrolment criteria were 29.7% aged ≥80 years, 62.4% weighing ≤60 kg, and 7.9% with serum creatinine ≥1.5 mg/dL. Of the cohort, 48.7% received on-label standard dose apixaban (5 mg twice daily), while 51.3% received off-label reduced dose (2.5 mg twice daily) (*Figure 1*).

### Baseline characteristics

Baseline characteristics of the on-label standard dose apixaban and off-label reduced-dose apixaban groups are presented in *Table 1*. In comparison with the on-label standard dose group, the off-label reduced-dose group exhibited older age and higher mean CHA_2_DS_2_–VASc and HAS-BLED scores. They had higher prevalence of hypertension, heart failure, prior stroke/TIA, prior bleeding, and CKD. This group was also more likely to receive concomitant antiplatelet therapy. Laboratory results showed greater prevalence of anaemia and CKD stage ≥3a with renal function impairment in the off-label reduced-dose group. No significant differences were observed in sex distribution or mean weight between groups. The standardized mean difference plot before and after IPTW is presented in Supplementary material online, *Figure S1*. After IPTW, baseline characteristics of the two groups were well-balanced (standardized mean differences ≤0.1) (Supplementary material online, *Table S2*).

**Table 1:** Baseline characteristics of the whole study population according to apixaban dose

		Apixaban dose		
	Total (*n* = 1944)	On-label standard dose (*n* = 947)	Off-label reduced dose (*n* = 997)	*P*-value
Single-dose reduction criteria				
Age ≥80 years	578 (29.7%)	242 (25.6%)	336 (33.6%)	<0.001
Body weight ≤60 kg	1213 (62.4%)	654 (69.1%)	559 (62.4%)	<0.001
Creatinine ≥1.5 mg/dL	153 (7.9%)	51 (5.4%)	102 (10.2%)	<0.001
Age (years)	74.3 ± 7.9	72.5 ± 8.2	76.0 ± 7.1	<0.001
<65	221 (11.4%)	150 (15.8%)	71 (7.1%)	<0.001
65–74	669 (34.4%)	397 (41.9%)	272 (27.3%)	<0.001
75–79	476 (24.5%)	158 (16.7%)	318 (31.9%)	<0.001
Sex (female)	1084 (55.8%)	533 (56.3%)	551 (55.3%)	0.652
Bodyweight (kg)	60.1 ± 10.0	59.8 ± 9.7	60.4 ± 10.3	0.073
≤50	247 (12.7%)	105 (11.1%)	142 (14.2%)	0.037
51–60	966 (49.7%)	549 (58.0%)	417 (41.8%)	<0.001
>60	731 (37.6%)	293 (30.9%)	438 (43.9%)	<0.001
CHA₂DS₂–VASc score	3.5 ± 1.4	3.4 ± 1.4	3.7 ± 1.3	<0.001
≥3	1494 (76.9%)	679 (71.7%)	815 (81.7%)	<0.001
HAS-BLED score^a^	1.6 ± 0.9	1.6 ± 0.9	1.7 ± 0.9	0.015
≥3	248 (12.8%)	113 (11.9%)	135 (13.5%)	0.209
Comorbidities				
Hypertension	1381 (71.0%)	652 (68.8%)	729 (73.1%)	0.038
Diabetes mellitus	608 (31.3%)	284 (30.0%)	324 (32.5%)	0.233
Heart failure	464 (23.9%)	201 (21.2%)	263 (26.4%)	0.008
Prior stroke/TIA	207 (10.6%)	125 (13.2%)	82 (8.2%)	<0.001
Prior bleeding	138 (7.1%)	50 (5.3%)	88 (8.8%)	0.002
CKD	213 (11.0%)	64 (6.8%)	149 (14.9%)	<0.001
Not on dialysis	173 (8.9%)	54 (5.7%)	119 (11.9%)	<0.001
On dialysis	25 (1.3%)	8 (0.8%)	17 (1.7%)	0.092
Previous kidney transplantation	14 (0.7%)	2 (0.2%)	12 (1.2%)	0.010
Liver disease	74 (3.8%)	33 (3.5%)	41 (4.1%)	0.470
Malignancy	265 (13.6%)	120 (12.7%)	145 (14.5%)	0.229
Antiplatelet use^b^	125 (6.6%)	44 (4.8%)	81 (8.4%)	0.002
SAPT	105 (5.6%)	36 (1.9%)	69 (3.7%)	0.624
DAPT	7 (0.4%)	4 (0.2%)	3 (0.2%)	0.211
Prior OAC	1444 (74.3%)	707 (74.7%)	737 (73.9%)	0.249
Prior VKA	54 (2.8%)	23 (2.4%)	31 (3.1%)	0.361
Prior DOAC	1390 (71.5%)	684 (72.2%)	706 (70.8%)	0.139
Apixaban	829 (42.6%)	437 (46.1%)	392 (39.3%)	0.001
Dabigatran	99 (5.1%)	51 (5.4%)	48 (4.8%)	0.561
Edoxaban	315 (16.2%)	131 (13.8%)	184 (18.5%)	0.005
Rivaroxaban	147 (7.6%)	65 (6.9%)	82 (8.2%)	0.253
Atrial fibrillation type				
Not determined	84 (4.3%)	31 (3.3%)	53 (5.3%)	0.027
Paroxysmal	1010 (52.0%)	499 (52.7%)	511 (51.3%)	0.526
Non-paroxysmal	850 (43.7%)	417 (44.0%)	433 (43.4%)	
Persistent	685 (35.2%)	325 (34.3%)	360 (36.1%)	0.055
Long-standing persistent	91 (4.7%)	48 (5.1%)	43 (4.3%)	0.456
Permanent	73 (3.8%)	43 (4.5%)	30 (3.0%)	0.078
EHRA classification				
I	460 (23.7%)	218 (23.0%)	242 (24.3%)	0.516
IIa	774 (39.8%)	379 (40.0%)	395 (39.6%)	0.856
IIb	236 (12.1%)	98 (10.3%)	138 (13.8%)	0.018
III	56 (2.9%)	20 (2.1%)	36 (3.6%)	0.048
IV	2 (0.1%)	1 (0.1%)	1 (0.1%)	0.971
Unknown	416 (21.4%)	231 (24.4%)	185 (18.6%)	0.002
Laboratory				
Haemoglobin (g/dL)^c^	13.0 ± 1.8	13.2 ± 1.8	12.8 ± 1.7	<0.001
(men ≤13 g/dL, female ≤12 g/dL)	523 (26.9%)	222 (23.4%)	301 (30.2%)	<0.001
Platelet (×10^3^/μL)	207.2 ± 69.0	209.1 ± 65.0	205.4 ± 72.6	0.175
PT INR^d^	1.2 ± 0.5	1.2 ± 0.3	1.2 ± 0.7	0.822
Creatinine (mg/dL)	1.0 ± 0.7	0.9 ± 0.6	1.1 ± 0.8	<0.001
CrCl (mL/min)	56.2 ± 17.5	60.1 ± 17.0	52.5 ± 17.1	<0.001
CrCl <50 (mL/min)	716 (36.8%)	271 (28.6%)	445 (44.6%)	<0.001
eGFR (MDRD) (mL/min/1.73 m^2^)	72.3 ± 23.1	76.2 ± 21.5	68.6 ± 24.0	<0.001
eGFR (CKD—EPI) (mL/min/1.73 m^2^)	71.0 ± 20.0	75.1 ± 18.3	67.0 ± 20.8	<0.001
Stage 1 (eGFR ≥90)	269 (13.8%)	180 (19.0%)	89 (8.9%)	<0.001
Stage 2 (60 ≤ eGFR < 90)	1086 (55.9%)	559 (59.0%)	527 (52.9%)	0.030
Stage 3a (45 ≤ eGFR < 60)	337 (17.3%)	135 (14.3%)	202 (20.3%)	<0.001
Stage 3b (30 ≤ eGFR < 45)	151 (7.8%)	54 (5.7%)	97 (9.7%)	0.001
Stage 4 (15 ≤ eGFR < 30)	42 (2.2%)	6 (0.6%)	36 (3.6%)	<0.001
Stage 5 (eGFR <15)	15 (0.8%)	3 (0.3%)	12 (1.2%)	0.023

Categorical variables were presented as a percentage and continuous variables were presented as mean and standard deviation.

CKD, chronic kidney disease; CrCl, creatinine clearance; DAPT, dual antiplatelet therapy; DOAC, direct oral anticoagulant; eGFR, estimated glomerular filtration rate; EHRA, European Heart Rhythm Association; EPI, Epidemiology Collaboration equation; MDRD, modification of diet in renal disease; PT INR, prothrombin time international normalized ratio; SAPT, single antiplatelet therapy; TIA, transient ischaemic attack; VKA, vitamin K antagonist.

^a^ *n* = 1881.

^b^ *n* = 1886.

^c^ *n* = 1726.

^d^ *n* = 520.

### Primary and secondary outcomes

**Figure 2:**
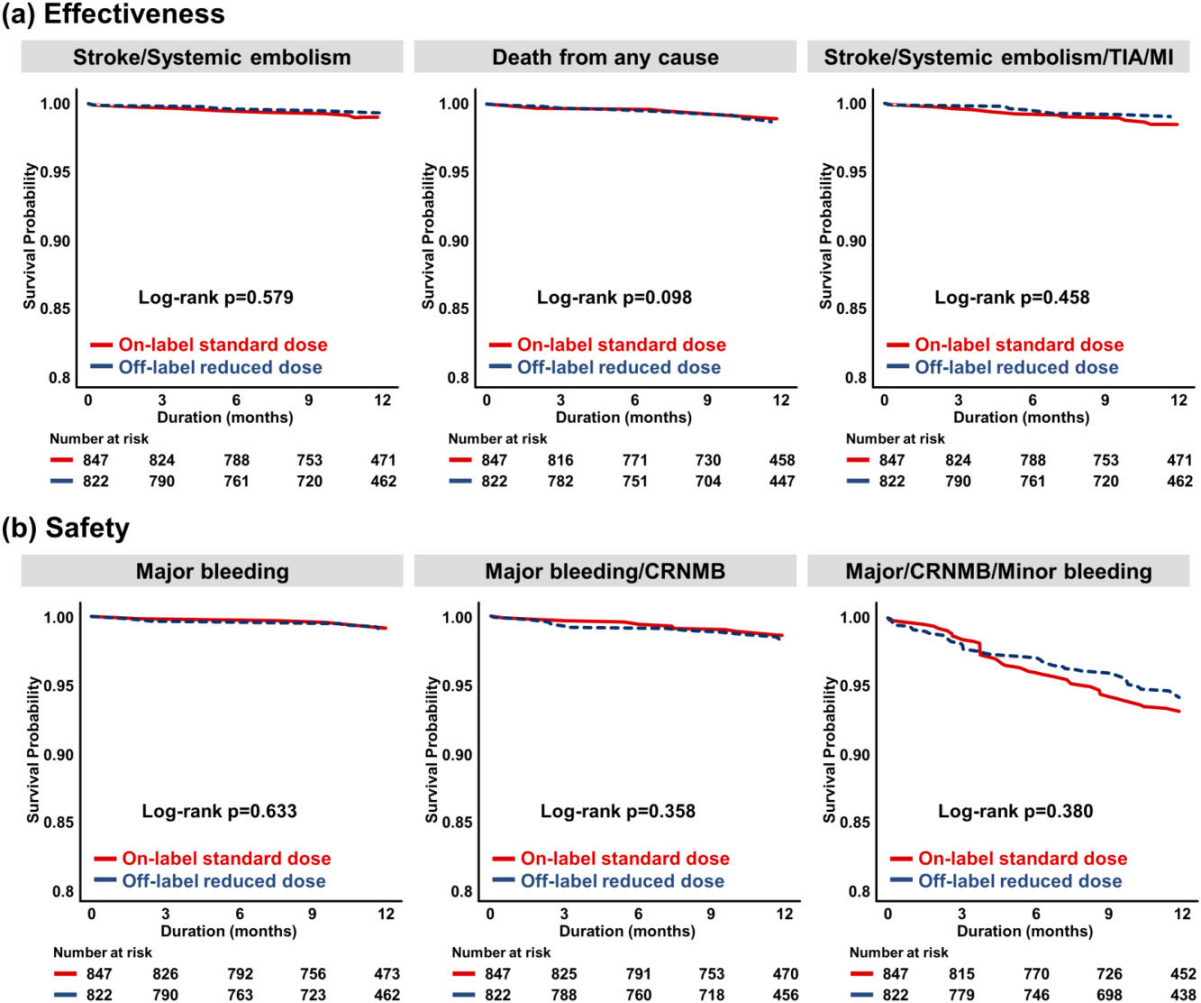
Kaplan–Meier curves of clinical outcomes after inverse probability of treatment weighting. After IPTW, Kaplan–Meier curves showed comparable risks for most outcomes, except for stroke/systemic embolism. Abbreviations: CRNMB, clinically relevant non-major bleeding; IPTW, inverse probability of treatment weighting; MI, myocardial infarction; TIA, transient ischaemic attack.

The crude IRs, unadjusted, adjusted, and weighted HRs, and Kaplan–Meier curves with log-rank *P* values of weighted IPTW population are presented in *Table 2*, Supplementary material online, *Table S3*, and *Figure 2*. During a mean follow-up period of 1.0 ± 0.2 years, the on-label standard dose group experienced eight SSE events compared with seven events in the off-label reduced-dose group (IR 0.9 vs. 0.7 per 100 PY). Major bleeding events were observed in 5 patients in the on-label standard dose group and 10 patients in the off-label reduced-dose group (IR 0.5 vs. 1.0 per 100 PY). The Kaplan–Meier curve of weighted IPTW population showed no statistically significant difference for both safety and effectiveness outcomes between the two dosage groups (all log-rank *P* > 0.05). Post-matching Cox proportional-hazards regression models with weights the off-label reduced-dose group demonstrated a comparable risk for SSE relative to the on-label standard dose group (HR 0.67, 95% CI 0.28–1.59, *P* = 0.370). Regarding secondary effectiveness outcomes, no significant differences were observed between the two groups after multivariable adjustment. Post-matching Cox proportional-hazards regression models with weights revealed no significant difference in the risk for major bleeding between off-label reduced-dose groups and the on-label standard dose group (HR 1.38, 95% CI 0.44–4.35, *P* = 0.578). For the secondary safety outcomes, no statistically significant differences were detected between the two groups following multivariable adjustment.

**Table 2:** Incident rate and hazard ratios of clinical outcomes

			**Events**	**IR per 100 PY**	**Unadjusted** **HR (95% CI)**	**Weighted** **HR (95% CI)**
Effectiveness	**Primary outcome: stroke/systemic embolism**					
	On-label standard dose		8	0.9	1.00 (reference)	1.00 (reference)
	Off-label reduced dose		7	0.7	0.84 (0.30–2.31)	0.67 (0.28–1.59)
	*P*-value				0.731	0.370
	**Secondary outcomes**					
	*TIA*					
	On-label standard dose		3	0.3	1.00 (reference)	1.00 (reference)
	Off-label reduced dose		1	0.1	0.32 (0.03–3.06)	0.42 (0.04–4.08)
	*P*-value				0.322	0.455
	*MI*					
	On-label standard dose		2	0.2	1.00 (reference)	1.00 (reference)
	Off-label reduced dose		2	0.2	1.04 (0.15–7.40)	1.31 (0.11–14.56)
	*P*-value				0.967	0.822
	*Death from cardiovascular cause*					
	On-label standard dose		2	0.2	1.00 (reference)	1.00 (reference)
	Off-label reduced dose		2	0.2	0.96 (0.14–6.82)	0.69 (0.09–5.01)
	*P*-value				0.967	0.714
	*Death from any cause*					
	On-label standard dose		5	0.5	1.00 (reference)	1.00 (reference)
	Off-label reduced dose		17	1.8	3.26 (1.20–8.84)	2.63 (0.93–7.45)
	*P*-value				0.020	0.068
	*Composite of stroke/systemic embolism/TIA/MI*					
	On-label standard dose		13	1.4	1.00 (reference)	1.00 (reference)
	Off-label reduced dose		10	1.1	0.73 (0.32–1.67)	0.67 (0.28–1.59)
	*P*-value				0.463	0.370
Safety	**Secondary outcomes: major bleeding**					
	On-label standard dose		5	0.5	1.00 (reference)	1.00 (reference)
	Off-label reduced dose		10	1.0	1.91 (0.65–5.58)	1.38 (0.44–4.35)
	*P*-value				0.238	0.578
	**Secondary outcomes**					
	*Major bleeding and clinically relevant non-major bleeding*					
	On-label standard dose		12	1.3	1.00 (reference)	1.00 (reference)
	Off-label reduced dose		21	2.2	1.67 (0.82–3.40)	1.55 (0.70–3.42)
	*P*-value				0.154	0.278
	*Composite of major, clinically relevant non-major bleeding, and minor bleeding*					
	On-label standard dose		57	6.4	1.00 (reference)	1.00 (reference)
	Off-label reduced dose		59	6.4	1.00 (0.69–1.43)	0.83 (0.52–1.33)
	*P*-value				0.966	0.451

CI, confidence interval; HR, hazard ratio; IR, incidence rate; MI, myocardial infarction; PY, person-years; TIA, transient ischaemic attack.

### Subgroup analysis

Subgroup analyses were conducted for pre-defined subgroups (see Supplementary material online, *Table S4*). No significant interactions were observed in subgroup analyses.

## Discussion

The key findings of this study are: (i) patients at high risk for bleeding (older age, higher comorbidity burden, and worse renal function) were more likely to receive off-label reduced-dose apixaban; and (ii) off-label reduced-dose apixaban showed no significant differences in effectiveness or safety compared with standard-dose apixaban after multivariable adjustment and IPTW. This study is the first prospective analysis to evaluate the safety and effectiveness of off-label reduced-dose apixaban in patients meeting a single-dose reduction criterion.

Off-label reduced-dose prescription in Asian patients with AF has been frequently observed across several studies.^7,9,17^ However, patient characteristics associated with this practice vary by region. In Taiwan, off-label underdosing was more common among younger patients with fewer comorbidities.^9^ Conversely, the Korean COmparison study of Drugs for symptom control and complication prEvention of AF (CODE-AF) registry found that off-label reduced dosing occurred in more than one-third of the population, particularly among older patients with lower body weight and reduced renal function.^7^

Our previous analysis of the ASPIRE cohort detailed baseline characteristics and factors linked to off-label reduced dosing.^18^ Patients at high risk for bleeding, especially those nearing a second dose reduction criterion, were more likely to receive reduced doses, while those with prior strokes were more often prescribed standard doses per labelling.^18^ These findings highlight the complexity of DOAC dosing in clinical practice. While established criteria provide a foundation for dosing decisions, our results indicate that clinicians often consider a broader range of patient characteristics when prescribing apixaban. This practice reflects the heterogeneity of the patient population and the need for individualized approaches to anticoagulation therapy.

In this study, the outcomes between the on-label standard dose group and off-label reduced-dose groups did not show any statistically significant differences when considering the baseline differences between the groups. The trend was similar to the previous study using Korean nationwide claims database, which showed higher risk of ischaemic stroke, all-cause death, and composite clinical outcomes but comparable risk of major bleeding in the off-label reduced apixaban dose group who already satisfied single-dose reduction criteria.^11^ Additionally, when comparing all four DOACs, underdosed patients did not show a significant difference in clinical outcomes from the labelled use group whereas overdosed patients showed worse clinical outcomes in Asian AF patients.^19^ In a meta-analysis on rivaroxaban dosing, J-ROCKET AF criteria of reduction showed comparable safety and effectiveness among Asian patients.^20^

These results, while differing from earlier studies reporting increased stroke risk and no major bleeding reduction in off-label dosing groups,^9,17,21^ suggest that outcomes may vary when patients are assessed based on adherence to dose reduction criteria. This underscores the importance of examining specific subgroups in off-label dosing studies. This aligns with findings from the ENGAGE AF-TIMI 48 trial, where the primary net clinical outcome improved with lower dosing, while secondary outcomes showed no significant differences between low and high doses.^22^ Considering the net clinical benefit, balancing stroke and bleeding risks may require a more individualized dosing approach, potentially diverging from standard on-label recommendations. Our findings further suggest that in patients at high risk for bleeding, lower doses may provide comparable net clinical benefits with reduced bleeding risks rather than avoiding oral anticoagulation entirely. Prospective registry data also indicate no significant differences in clinical outcomes between on-label and off-label dosing.

The ARISTOTLE trial established apixaban's official dosing protocol, using a standard dose of 5 mg twice daily and a reduced dose of 2.5 mg twice daily for patients meeting at least two specific criteria, which demonstrated superior stroke prevention and reduced bleeding compared with warfarin.^5^ In the ARISTOTLE trial, patients with a single-dose reduction criterion accounted for 22.8% of the total study population. These patients generally had a higher risk of bleeding and thrombo-embolic events compared with those with no dose reduction criteria. However, even in this subgroup, apixaban 5 mg twice daily demonstrated similar benefits to the main ARISTOTLE results, showing superiority over warfarin in reducing stroke, major bleeding, and intracranial haemorrhage.^23^ In the ARISTOTLE trial, patients in this subgroup were not included in the 2.5 mg twice daily regimen, making it impossible to infer its clinical outcomes. Unlike ARISTOTLE, our study evaluated the clinical outcomes of patients meeting a single-dose reduction criterion who were prescribed either apixaban 5 mg or 2.5 mg twice daily at the physician's discretion. Our findings indicate no significant difference in ischaemic or bleeding outcomes compared with the on-label dose in these single-dose reduction criterion patients, suggesting that some individuals may tolerate a lower dose without compromising efficacy or safety. Although this study is not a randomized controlled trial and cannot conclusively recommend dose reduction for patients meeting the single-dose reduction criterion, it highlights the need for individualized assessment in specific populations without overriding existing guidelines.

Monitoring DOAC plasma concentrations may help determine appropriate individualized dosing strategies for patients at high risk for bleeding. While off-label reduced doses typically result in lower plasma concentrations, the relationship between drug levels and clinical outcomes is complex.^24,25^ The ENGAGE AF-TIMI 48 trial showed that Asian patients, often with lower body weight, had reduced edoxaban concentrations and anti-factor Xa activity but a higher bleeding risk.^25^ Another study found that some patients on off-label underdosed apixaban or rivaroxaban still achieved acceptable peak plasma concentrations.^26^ These findings highlight the intricate relationship between drug levels and therapeutic outcomes, supporting the need for individualized DOAC dosing, potentially with pharmacokinetic monitoring.

### Limitations

This study has certain limitations that should be acknowledged. First, our study was conducted at a single tertiary medical centre in Korea with a predominantly Asian patient population, which may limit the generalizability of our findings to other racial or ethnic groups and different healthcare systems. Previous research has shown that there can be racial differences in both stroke and bleeding outcomes, highlighting the need for caution when extrapolating these results to more diverse or non-Asian populations.^27,28^ Second, the relatively short mean follow-up period may not fully capture long-term outcomes or rare events in this chronic condition, limiting the generalizability of the findings to long-term anticoagulation management. To address this limitation, we are planning an extended cohort analysis with a longer follow-up duration, providing more comprehensive insights into the long-term effectiveness and safety of different apixaban dosing strategies. Third, despite the use of IPTW and multivariable adjustments, unmeasured confounders may still influence the results due to the study's observational design. Our dataset did not capture the duration of apixaban use prior to enrolment, including potential dose adjustments made for clinical reasons before study initiation. Additionally, changes in apixaban dose or other medications, including antiplatelet therapy during follow-up, may have also impacted the findings. Such data were not collected or included in this study and, therefore, could not be adjusted for. This should be considered when interpreting the results. Fourth, the small number of events, particularly for primary outcomes, may have reduced the statistical power to detect significant differences. During the follow-up period, we observed only 15 SSE (8 in the on-label group and 7 in the off-label group) and 15 major bleeding events (5 in the on-label group and 10 in the off-label group). The confidence intervals in our results suggest some degree of uncertainty, which is not uncommon in studies with a limited number of events. Fifth, data on patient adherence to the prescribed apixaban regimen were unavailable, potentially affecting the interpretation of effectiveness and safety outcomes. Sixth, the findings are specific to apixaban and may not be generalizable to other DOACs. Lastly, the lack of pharmacokinetic data limits the interpretation of the relationship between dosing, drug levels, and clinical outcomes.

## Conclusion

In this prospective cohort of Korean AF patients meeting a single-dose reduction criterion, off-label reduced-dose apixaban was not associated with a higher risk of SSE or major bleeding compared with on-label standard dosing. These findings suggest the potential value of individualized anticoagulation strategies (e.g. reduced-dose apixaban), particularly for patients at high risk for bleeding. Further research, including randomized controlled trials, is warranted to investigate alternative apixaban dosing strategies for patients at high risk of bleeding, which may be particularly relevant for East Asian populations.

## Supplementary Material

pvaf018_Supplemental_File

## Notes

**Conflict of interest:** S.‑R.L.: speaking fees from Bayer, BMS/Pfizer, Biosense Webster, Daiichi-Sankyo, Sanofi-Aventis, Daewoong Pharmaceutical Co., Samjinpharm, Seers Technology, Biotronik, Boston Scientific, and Medtronic and is a consultant for Biosense Webster. E.-K.C.: research grants or speaking fees from Abbott, Bayer, BMS/Pfizer, Biosense Webster, Chong Kun Dang, Daewoong Pharmaceutical Co., Daiichi-Sankyo, DeepQure, Dreamtech Co., Ltd, Jeil Pharmaceutical Co. Ltd, Medtronic, Samjinpharm, Samsung Electronics Co., Ltd, Seers Technology, and Skylabs. G.Y.H.L.: consultant and speaker for BMS/Pfizer, Boehringer Ingelheim, Anthos, and Daiichi-Sankyo. No fees are received personally. All other authors have reported that they have no relationships relevant to the contents of this paper to disclose.

## Data availability

The data underlying this article will be shared on reasonable request to the corresponding author.

## Notes

A complete list of the ASPIRE investigators is provided in the Supplemental Material (see Supplementary material online, *Table S1*).
