# Supplementary material for: Apixaban outcomes in atrial fibrillation patients with a single-dose reduction criterion: ASPIRE 1-year results
Source: Eur Heart J Cardiovasc Pharmacother. 2025 Mar 20;11(5):403–11. doi: 10.1093/ehjcvp/pvaf018 (PMC12343049; doi:10.1093/ehjcvp/pvaf018)
Supplement: pvaf018_Supplemental_File [file pvaf018_supplemental_file.docx]

**Supplemental Materials**

**Supplemental Methods**

**Supplemental Tables**

Table 1. ASPIRE sites, investigators, and affiliations.

Table 2. Baseline characteristics before and after IPTW

Table 3. Incident rate and hazard ratios of clinical outcomes

Table 4. Subgroup analysis

**Supplemental Figures**

Figure 1. Standardized mean differences before and after inverse probability of treatment weighting

**Supplemental References**

**Supplemental Methods**

The ASPIRE (Efficacy and Safety of Apixaban in Real-world Practice in Korean Frail Patients with Atrial Fibrillation) study was a prospective, multicenter, non-interventional observational investigation encompassing all geographic areas within South Korea across 32 participating centers. The protocols received approval from the ethics committees of each center and were carried out in adherence to the principles outlined in the Declaration of Helsinki (H-2108-110-1245). This study was registered on ClinicalTrials.gov (NCT05773222). Informed consent was obtained from all patients before their inclusion in the study.

*Study population and design*

The details of the study protocol are described in our previous report.(1) Briefly, participants aged 19 years or older who had non-valvular AF and were prescribed apixaban, as well as those meeting the single dose criterion for apixaban, were screened. The criteria for apixaban dose reduction included: (1) age 80 years or older, (2) body weight 60 kg or less, and (3) serum creatinine level 1.5 mg/dL or higher.(2) Exclusion criteria comprised: (1) vulnerability (as defined by Korean Good Clinical Practice) or disagreement with the study, (2) patients who experienced clinical events, as outlined in the primary and secondary outcomes of the study, prior to enrollment while on apixaban, and (3) meeting two or more dose reduction criteria for apixaban.

Among these eligible participants, the choice of specific apixaban dosage – either 5 mg twice daily (on-label standard dose apixaban) or 2.5 mg twice daily (off-label reduced dose apixaban) – was left to the discretion of the treating physicians.

*Covariates*

Demographic details and anthropometric measurements, encompassing age, gender, weight, height, and body mass index, were recorded. Additionally, systolic and diastolic blood pressure, along with heart rate, were documented. Baseline variables comprised comorbidities such as hypertension, diabetes mellitus, heart failure, history of stroke/transient ischemic attack (TIA), bleeding, chronic kidney disease (CKD), liver disease, and malignancy. Laboratory findings encompassed complete blood count (hemoglobin and platelets), prothrombin time, international normalized ratio (INR), and chemistry parameters (creatinine, creatinine clearance [CrCl], and estimated glomerular filtration rate [eGFR] with the Modification of Diet in Renal Disease [MDRD] equation and CKD Epidemiology Collaboration [CKD-EPI] equation). CHA_2_DS_2_-VASc and HAS-BLED scores were computed based on participants’ comorbidities and laboratory results.(3) The diagnosis of AF included information on its type (paroxysmal, non-paroxysmal [persistent, long-standing persistent, permanent]), European Heart Rhythm Association (EHRA) symptom classification, and rhythm control status. Pharmacological treatment data at baseline included OAC history including specific types of OAC, and concomitant use of antiplatelet therapy (APT). Anemia was defined as hemoglobin 13 g/dL or less for men and hemoglobin 12 g/dL or less for women.

*Follow-up and outcomes*

The study was prospectively followed up to 12 months after enrollment, and the recommended schedule for data collection was every 3 months. However, this was at the discretion of the physician. The primary effectiveness outcome was defined as the occurrence of the first stroke/systemic embolism during follow-up; secondary effectiveness outcomes were defined as TIA, myocardial infarction, death from cardiovascular cause, death from any cause, and a composite of thromboembolic events including stroke/systemic embolism, TIA, and myocardial infarction. The primary safety outcome was defined as the occurrence of the first major bleeding according to the criteria of the International Society on Thrombosis and Haemostasis (ISTH) and secondary safety outcomes were defined as a composite of major bleeding and clinically relevant non-major bleeding (CRNMB), and a composite of major bleeding, CRNMB, and minor bleeding.(4) During follow-up, apixaban dosing status and dose changes were collected, and laboratory test follow-up at 12-month was recommended.

*Statistical methods*

Regarding baseline characteristics, continuous variables are expressed as mean ± standard deviation, while categorical variables are presented as numbers and percentages. Group comparisons were conducted using various statistical tests including the Mann-Whitney U, chi-square, Analysis of Variance, and Fisher’s exact tests.

The incidence rates (IRs) were determined by dividing the number of events occurring during the follow-up period by the 100 person-year (PY) risk. Survival analysis using the Kaplan-Meier method (log-rank test) and Cox proportional hazards regression model were employed to evaluate the risks of primary and secondary outcomes in between the off-label low-dose apixaban group and the on-label standard dose apixaban group (reference). Hazard ratios (HRs) along with 95% confidence intervals (CIs) are presented, with adjustments made in stepwise manner: model 1 was unadjusted, model 2 was adjusted for age and sex, model 3 was adjusted for age, sex, hypertension, diabetes mellitus, heart failure, CKD, previous stroke or TIA, previous bleeding, and malignancy, while model 4 further adjusted for concomitant APT use and anemia as in model 3. To compare the clinical outcomes between the two groups after balancing the differences of baseline characteristics of the two groups, we performed an inverse probability of treatment weighting (IPTW) analysis.(5) The matching variables included age, sex, hypertension, diabetes, heart failure, CKD, history of stroke/TIA, history of bleeding, malignancy, APT, and anemia. Propensity scores were generated using logistic regression, and group proportions were weighted to create stabilized IPTW for inclusion in the final analysis.(5, 6) Differences between pre- and post-matched variables were assessed using standardized mean differences, with a value of 0.1 or less indicating that the groups were well-balanced on that variable, making the difference negligible.(7) For the post-matching survival analysis, weighted Log-rank tests and Cox proportional hazards regression models were employed to account for the calculated weights.(6)

Statistical significance was defined as p< 0.05. All statistical analyses were conducted utilizing SPSS version 25 (IBM Corp., Armonk, NY, USA).

*Subgroup analysis*

Subgroup analyses were performed to determine the effectiveness (SSE and death from any cause) and primary safety outcomes. We investigated the clinical outcomes of on-label standard dose apixaban compared to off-label reduced dose apixaban across age categories (≥80 years, <80 years), body weight (≤60 kg, >60 kg), and serum creatinine levels (≥1.5 mg/dL, <1.5 mg/dL), in accordance with each component of the apixaban dose reduction criterion. Subgroup analyses were also performed based on CHA_2_DS_2_-VASc score (<2, ≥2), HAS-BLED score (<2, ≥2), and CKD-EPI eGFR (≥60 mL/min/1.73m^2^, <60 mL/min/1.73m^2^). Subgroup analyses were conducted utilizing a multivariable Cox proportional hazards regression model incorporating adjustments from model 4.

*Sample Size Calculation*

Assuming an incidence of outcomes (stroke or systemic embolism/bleeding) of ≤4.5% in a high-risk population characterized by advanced age, low body weight, and impaired renal function, a sample size of 734 participants per subgroup was required to ensure an estimated precision of 1.5%. To account for an anticipated dropout rate of approximately 12%, we planned to recruit 834 participants in each subgroup: those aged ≥80 years, those with a body weight ≤60 kg, and those with impaired renal function (serum creatinine levels ≥1.5 mg/dL). This yielded a total planned sample size of 2,500 participants. However, due to delays in enrollment caused by the COVID-19 pandemic, the protocol was amended to target a reduced study population of 2,000 participants.

**Table S1. ASPIRE sites, investigators, and affiliations**

| **Site** | **Investigator names and academic degree** | **Affiliations** |
| --- | --- | --- |
| Gangsim Heart Clinic | Seongwook Han, MD, PhD | Division of Cardiology, Department of Internal Medicine, Keimyung University Dongsan Hospital, Daegu |
| Kyung Hee University Hospital at Gangdong | Eun-Sun Jin, MD, PhD | Department of Cardiology, Kyung Hee University College of Medicine, Kyung Hee University Hospital at Gangdong, Seoul |
| Kangbuk Samsung Hospital | Sung Ho Lee, MD, PhD | Division of Cardiology, Department of Internal Medicine, Kangbuk Samsung Hospital, Sungkyunkwan University School of Medicine, Seoul |
| Kyung-Hee University Hospital | Jin-Bai Kim, MD, PhD | Division of Cardiology, Department of Internal Medicine, Kyung-Hee University Hospital, Kyung-Hee University, Seoul |
| Keimyung University Dongsan Hospital | Hyoung-Seob Park, MD | Division of Cardiology, Department of Internal Medicine, Keimyung University Dongsan Hospital, Daegu |
|  | Jongmin Hwang, MD, PhD | Division of Cardiology, Department of Internal Medicine, Keimyung University Dongsan Hospital, Daegu |
| Korea University Guro Hospital | Seung-Young Roh, MD, PhD | Department of Internal Medicine, Korea University College of Medicine, Seoul |
| Korea University Anam Hospital | Jong-Il Choi, MD, PhD, MHS, MS | Department of Internal Medicine, Division of Cardiology, Korea University College of Medicine and Korea University Anam Hospital, Seoul |
| Kosin University Gospel Hospital | Jung Ho Heo, MD, PhD | Department of Internal Medicine, Kosin University Gospel Hospital, Busan |
| National Health Insurance Service Ilsan Hospital | Jeon Dong Woon, MD, PhD | Department of Internal Medicine, National Health Insurance Service Ilsan Hospital, Gyeonggi |
| Dongguk University Ilsan Hospital | Ungjeong Do, MD | Division of Cardiology, Department of Internal Medicine, Dongguk University Ilsan Hospital, Goyang, Korea |
| Dong-A University Hospital | Jong-Sung Park, MD, PhD | Department of Cardiology, Dong-A University Hospital, Busan |
|  | Kyung Hee Lim, MD, PhD | Department of Cardiology, Dong-A University Hospital, Busan |
| Seoul National University Hospital | Eue-Keun Choi, MD, PhD | Department of Internal Medicine, Seoul National University Hospital, Seoul |
|  | So‑Ryoung Lee, MD, PhD | Department of Internal Medicine, Seoul National University Hospital, Seoul |
|  | Seil Oh, MD, PhD | Department of Internal Medicine, Seoul National University Hospital, Seoul |
| Seoul National University Bundang Hospital | Il-Young OH, MD, Ph.D | Department of Internal Medicine, Seoul National University Bundang Hospital, Gyeonggi |
|  | Cho, Youngjin, MD, PhD | Division of Cardiology, Seoul National University Bundang Hospital, Gyeonggi |
|  | Lee, Jihyun, MD, PhD | Division of Cardiology, Seoul National University Bundang Hospital, Gyeonggi |
| Samsung Medical Center | Young Keun On, MD, PhD | Department of Cardiology, Heart Vascular Stroke Institute, Samsung Medical Center, Sungkyunkwan University School of Medicine, Seoul |
| Samsung Changwon Hospital | Hye Bin Gwag, MD, PhD | Department of Internal Medicine, Samsung Changwon Hospital, Sungkyunkwan University School of Medicine, Changwon |
| SMG-SNU Boramae Medical Center | Woo-Hyun Lim, MD | Department of Internal Medicine, SMG-SNU Boramae Medical Center, Seoul |
| Asan Medical Center | Kee-Joon Choi, MD, PhD | Department of Internal Medicine, Asan Medical Center, Ulsan University, Seoul |
| Soonchunhyang University Cheonan Hospital | Seung-Jin Lee, MD, PhD | Department of Internal Medicine, Soonchunhyang University Cheonan Hospital, Cheonan |
| Severance Cardiovascular Hospital | Hee Tae Yu, MD, PhD | Department of Internal Medicine, Severance Cardiovascular Hospital, Yonsei University College of Medicine, Seoul |
|  | Tae-Hoon Kim, MD | Department of Internal Medicine, Severance Cardiovascular Hospital, Yonsei University College of Medicine, Seoul |
|  | Daehoon Kim, MD | Department of Internal Medicine, Severance Cardiovascular Hospital, Yonsei University College of Medicine, Seoul |
| Ajou University Hospital | Kwang-No Lee, MD, PhD | Department of Cardiology, Ajou University School of Medicine, Suwon |
| Andong General Hospital | Dae-Woo Hyun, MD, PhD | Department of Cardiovascular Center, Andong General Hospiral, Andong |
| Yeungnam University Hospital | Dong-Gu Shin, MD, PhD | Department of Cardiovascular Division, Yeungnam University Hospital, Daegu |
| Wonkwang University Hospital | Nam-Ho Kim, MD, PhD | Department of Internal Medicine, Wonkwang University Hospital, Iksan |
|  | Kyeong Ho Yun, MD, PhD | Department of Internal Medicine, Wonkwang University Hospital, Iksan |
| Inje University Haeundae Paik Hospital | Sang-Hoon Seol, MD, PhD | Department of Internal Medicine, Inje University Haeundae Paik Hospital, Busan |
| Inje University Pusan Paik Hospital | Dae-Kyeong Kim, MD, PhD | Department of Internal Medicine, Inje University Pusan Paik Hospital, Busan |
| Inje University Ilsan Paik Hospital | June Namgung, MD, PhD | Department of Internal Medicine, Inje University Ilsan Paik Hospital, Goyang-si |
| Chonnam National University Hospital | Hyung Wook Park, MD, PhD | Department of Cardiovascular Medicine, Chonnam National University Hospital, Gwangju |
| Chonnam National University Hospital | Ki Hong Lee, MD, PhD, FESC, FKHRS, | Department of Cardiovascular medicine, Chonnam National University Hospital, Gwangju |
| Jeonbuk National University Hospital | Kyoung-Suk Rhee, MD, PhD | Department of Internal Medicine, Jeonbuk National University Hospital, Jeonbuk |
| Jeju National University hospital | Joon Hyouk Choi, MD, PhD | Department of Cardiology, Jeju national university hospital, Jeju |
| Chung-Ang University Hospital | Seung Yong Shin, MD, PhD | Cardiovascular & Arrhythmia center, Chung-Ang University Hospital, Seoul |
| Hanyang University Guri Hospital | Hwan-Cheol Park MD, PhD | Division of Cardiology, Department of Internal Medicine, Hanyang University College of Medicine, Gyeonggi |
| Hanyang University Medical Center | Jin-Kyu Park, MD, PhD | Department of Internal Medicine, Hanyang University Medical Center, Seoul |

**Table S2. Baseline characteristics before and after IPTW**

| **Variables** | **On-label standard dose** | **Off-label reduced dose** | **SMD** | **On-label standard dose** | **Off-label reduced dose** | **SMD** |
| --- | --- | --- | --- | --- | --- | --- |
| **N** | 847 | 822 |  | 860.61 | 819.12 |  |
| **Age, years (mean ± SD)** | 72.35 ± 8.17 | 75.86 **±** 7.30 | 0.453 | 74.26 ± 7.88 | 73.97 ± 8.42 | 0.035 |
| **Sex (Male)** | 367 (43.3%) | 378 (46.0%) | 0.053 | 397.2 (46.2%) | 373.4 (45.6%) | 0.011 |
| **Hypertension** | 586 (69.2%) | 614 (74.7%) | 0.123 | 623.9 (72.5%) | 589.3 (71.9%) | 0.012 |
| **Diabetes Mellitus** | 254 (30.0%) | 262 (31.9%) | 0.041 | 258.1 (30.0%) | 251.1 (30.7%) | 0.014 |
| **Heart failure** | 174 (20.5%) | 225 (27.4%) | 0.161 | 216.5 (25.2%) | 202.1 (24.7%) | 0.011 |
| **Chronic Kidney disease** | 58 (6.8%) | 131 (15.9%) | 0.289 | 113.5 (13.2%) | 95.6 (11.7%) | 0.046 |
| **Prior stroke/TIA** | 118 (13.9%) | 69 (8.4%) | 0.177 | 92.6 (10.8%) | 89.9 (11.0%) | 0.007 |
| **Prior bleeding** | 42 (5.0%) | 65 (7.9%) | 0.120 | 59.1 (6.9%) | 57.2 (7.0%) | 0.004 |
| **Malignancy** | 107 (12.6%) | 120 (14.6%) | 0.057 | 128.2 (14.9%) | 109.8 (13.4%) | 0.043 |
| **Antiplatelet use** | 41 (4.8%) | 74 (9.0%) | 0.165 | 63.9 (7.4%) | 56.7 (6.9%) | 0.020 |
| **Anemia** | 216 (25.5%) | 289 (35.2%) | 0.211 | 277.2 (32.2%) | 254.9 (31.1%) | 0.023 |

Abbreviation: IPTW, inverse probability of treatment weighting; SD, standard deviation; SMD, standardized mean difference; TIA, transient ischemic attack.

**Table S3. Incident rate and hazard ratios of clinical outcomes**

|  |  |  | **Events** | **IR per 100 PY** | **Unadjusted**  **HR (95% CI)** | | **Adjusted**  **HR* (95% CI)** |
| --- | --- | --- | --- | --- | --- | --- | --- |
| **Effectiveness** | **Primary outcome: Stroke/systemic embolism** | | | | | | |
|  | **On-label standard dose** | | 8 | 0.9 | 1.00 (reference) | | 1.00 (reference) |
|  | **Off-label reduced dose** | | 7 | 0.7 | 0.84 (0.30–2.31) | | 0.66 (0.23–1.90) |
|  | **p-value** | | | | 0.731 | | 0.439 |
|  | **Secondary outcomes** | | | | |  | |
|  | **TIA** | | | | | | |
|  | **On-label standard dose** | | 3 | 0.3 | 1.00 (reference) | | 1.00 (reference) |
|  | **Off-label reduced dose** | | 1 | 0.1 | 0.32 (0.03–3.06) | | 0.32 (0.03–3.51) |
|  | **p-value** | | | | 0.322 | | 0.351 |
|  | **MI** | | | | |  | |
|  | **On-label standard dose** | | 2 | 0.2 | 1.00 (reference) | | 1.00 (reference) |
|  | **Off-label reduced dose** | | 2 | 0.2 | 1.04 (0.15–7.40) | | 0.67 (0.05–9.31) |
|  | **p-value** | | | | 0.967 | | 0.768 |
|  | **Death from cardiovascular cause** | | | | | | |
|  | **On-label standard dose** | | 2 | 0.2 | 1.00 (reference) | | 1.00 (reference) |
|  | **Off-label reduced dose** | | 2 | 0.2 | 0.96 (0.14–6.82) | | 0.30 (0.03–3.25) |
|  | **p-value** | | | | 0.967 | | 0.320 |
|  | **Death from any cause** | | | | | | |
|  | **On-label standard dose** | | 5 | 0.5 | 1.00 (reference) | | 1.00 (reference) |
|  | **Off-label reduced dose** | | 17 | 1.8 | 3.26 (1.20–8.84) | | 2.35 (0.83–6.67) |
|  | **p-value** | | | | **0.020** | | 0.109 |
|  | **Composite of stroke/systemic embolism/TIA/MI** | | | | | | |
|  | **On-label standard dose** | | 13 | 1.4 | 1.00 (reference) | | 1.00 (reference) |
|  | **Off-label reduced dose** | | 10 | 1.1 | 0.73 (0.32–1.67) | | 0.62 (0.26–1.51) |
|  | **p-value** | | | | 0.463 | | 0.294 |
| **Safety** | **Secondary outcomes: Major bleeding** | | | | |  | |
|  | **On-label standard dose** | | 5 | 0.5 | 1.00 (reference) | | 1.00 (reference) |
|  | **Off-label reduced dose** | | 10 | 1.0 | 1.91 (0.65–5.58) | | 1.13 (0.35–3.70) |
|  | **p-value** | | | | 0.238 | | 0.838 |
|  | **Secondary outcomes** | | | | |  | |
|  | **Major bleeding or clinically relevant nonmajor bleeding** | | | | | | |
|  | **On-label standard dose** | | 12 | 1.3 | 1.00 (reference) | | 1.00 (reference) |
|  | **Off-label reduced dose** | | 21 | 2.2 | 1.67 (0.82–3.40) | | 1.31 (0.58–2.94) |
|  | **p-value** | | | | 0.154 | | 0.504 |
|  | **Composite of major, clinically relevant nonmajor bleeding, and minor bleeding** | | | | | | |
|  | **On-label standard dose** | | 57 | 6.4 | 1.00 (reference) | | 1.00 (reference) |
|  | **Off-label reduced dose** | | 59 | 6.4 | 1.00 (0.69–1.43) | | 0.88 (0.57–1.34) |
|  | **p-value** | | | | 0.966 | | 0.548 |

*Adjusted for age, sex, hypertension, diabetes mellitus, heart failure, CKD, prior stroke or TIA, prior bleeding, malignancy, antiplatelet use, anemia

Abbreviation: CI, confidence interval; HR, hazard ratio; IR, incidence rate; MI, myocardial infarction; PY, person-years; TIA, transient ischemic attack.

**Table S4. Subgroup analysis**

|  | | | **IR per 100 PY** | **Adjusted HR* (95% CI)** | **p-value** | **p-for-interaction** |
| --- | --- | --- | --- | --- | --- | --- |
| **Age** | | | | | | |
| **Effectiveness** | **Primary outcome: Stroke/systemic embolism** | | | | | |
|  | **≥80 years** | **On-label standard dose** | 1.3 | 1 (references) | 0.585 | 0.974 |
|  |  | **Off-label reduced dose** | 0.9 | 0.62 (0.11-3.41) |  |  |
|  | **<80 years** | **On-label standard dose** | 0.7 | 1 (references) | 0.440 |  |
|  |  | **Off-label reduced dose** | 0.6 | 0.57 (0.14-2.36) |  |  |
|  | **Secondary outcomes** | | | | | |
|  | **Death from any cause** | | | | | |
|  | **≥80 years** | **On-label standard dose** | 0.8 | 1 (references) | 0.730 | 0.236 |
|  |  | **Off-label reduced dose** | 1.6 | 1.36 (0.23-7.92) |  |  |
|  | **<80 years** | **On-label standard dose** | 0.4 | 1 (references) | 0.254 |  |
|  |  | **Off-label reduced dose** | 1.9 | 2.22 (0.56-8.77) |  |  |
| **Safety** | **Primary outcome: Major bleeding** | | | | | |
|  | **≥80 years** | **On-label standard dose** | 1.3 | 1 (references) | 0.485 | 0.506 |
|  |  | **Off-label reduced dose** | 0.6 | 0.51 (0.07-3.33) |  |  |
|  | **<80 years** | **On-label standard dose** | 0.3 | 1 (references) | 0.344 |  |
|  |  | **Off-label reduced dose** | 1.3 | 2.42 (0.38-15.18) |  |  |
| **Body weight** | | | | | | |
| **Effectiveness** | **Primary outcome: Stroke/systemic embolism** | | | | | |
|  | **≤60 kg** | **On-label standard dose** | 0.8 | 1 (references) | 0.400 | 0.461 |
|  |  | **Off-label reduced dose** | 0.8 | 0.53 (0.12-2.31) |  |  |
|  | **>60 kg** | **On-label standard dose** | 1.1 | 1 (references) | 0.520 |  |
|  |  | **Off-label reduced dose** | 0.7 | 0.58 (0.11-3.03) |  |  |
|  | **Secondary outcomes:** | | | | | |
|  | **Death from any cause** | | | | | |
|  | **≤60 kg** | **On-label standard dose** | 0.5 | 1 (references) | 0.473 | 0.430 |
|  |  | **Off-label reduced dose** | 1.7 | 1.69 (0.40-7.08) |  |  |
|  | **>60 kg** | **On-label standard dose** | 0.7 | 1 (references) | 0.382 |  |
|  |  | **Off-label reduced dose** | 1.9 | 2.04 (0.41-10.24) |  |  |
| **Safety** | **Primary outcome: Major bleeding** | | | | | |
|  | **≤60 kg** | **On-label standard dose** | 0.3 | 1 (references) | 0.434 | 0.152 |
|  |  | **Off-label reduced dose** | 1.1 | 2.28 (0.28-18.04) |  |  |
|  | **>60 kg** | **On-label standard dose** | 1.1 | 1 (references) | 0.560 |  |
|  |  | **Off-label reduced dose** | 1.0 | 0.62 (0.12-3.05) |  |  |
| **Serum creatinine levels** | | | | | | |
| **Effectiveness** | **Primary outcome: Stroke/systemic embolism** | | | | | |
|  | **≥1.5 mg/dL** | **On-label standard dose** | 0.0 | 1 (references) | - | <0.001 |
|  |  | **Off-label reduced dose** | 0.0 | - |  |  |
|  | **<1.5 mg/dL** | **On-label standard dose** | 0.9 | 1 (references) | 0.537 |  |
|  |  | **Off-label reduced dose** | 0.8 | 0.72 (0.25 – 2.07) |  |  |
|  | **Secondary outcomes** | | | | | |
|  | **Death from any cause** | | | | | |
|  | **≥1.5 mg/dL** | **On-label standard dose** | 0.0 | 1 (references) | 0.976 | 0.567 |
|  |  | **Off-label reduced dose** | 3.1 | 114334.73 (4.10E^-322^-) |  |  |
|  | **<1.5 mg/dL** | **On-label standard dose** | 0.6 | 1 (references) | 0.288 |  |
|  |  | **Off-label reduced dose** | 1.6 | 1.81 (0.60-5.41) |  |  |
| **Safety** | **Primary outcome: Major bleeding** | | | | | |
|  | **≥1.5 mg/dL** | **On-label standard dose** | 0.0 | 1 (references) | 0.981 | 0.333 |
|  |  | **Off-label reduced dose** | 2.0 | 133821.23 (0.00-) |  |  |
|  | **<1.5 mg/dL** | **On-label standard dose** | 0.6 | 1 (references) | 0.825 |  |
|  |  | **Off-label reduced dose** | 0.9 | 1.15 (0.32-4.08) |  |  |
| **CHA_2_DS_2_-VASc score** | | | | | | |
| **Effectiveness** | **Primary outcome: Stroke/systemic embolism** | | | | | |
|  | **<2** | **On-label standard dose** | 0.0 | 1 (references) | - | <0.001 |
|  |  | **Off-label reduced dose** | 0.0 | - |  |  |
|  | **≥2** | **On-label standard dose** | 0.9 | 1 (references) | 0.425 |  |
|  |  | **Off-label reduced dose** | 0.7 | 0.65 (0.23 – 1.87) |  |  |
|  | **Secondary outcome** | | | | | |
|  | **Death from any cause** | | | | | |
|  | **<2** | **On-label standard dose** | 0.0 | 1 (references) | - | 0.996 |
|  |  | **Off-label reduced dose** | 0.0 | - |  |  |
|  | **≥2** | **On-label standard dose** | 0.6 | 1 (references) | 0.189 |  |
|  |  | **Off-label reduced dose** | 1.8 | 2.03 (0.70-5.89) |  |  |
| **Safety** | **Primary outcome: Major bleeding** | | | | | |
|  | **<2** | **On-label standard dose** | 0.0 | 1 (references) | - | 0.987 |
|  |  | **Off-label reduced dose** | 0.0 | - |  |  |
|  | **≥2** | **On-label standard dose** | 0.6 | 1 (references) | 0.861 |  |
|  |  | **Off-label reduced dose** | 1.1 | 1.11 (0.34-3.63) |  |  |
| **HAS-BLED score** | | | | | | |
| **Effectiveness** | **Primary outcome: Stroke/systemic embolism** | | | | | |
|  | **<2** | **On-label standard dose** | 1.4 | 1 (references) | 0.118 | 0.427 |
|  |  | **Off-label reduced dose** | 0.5 | 0.26 (0.05 – 1.40) |  |  |
|  | **≥2** | **On-label standard dose** | 0.4 | 1 (references) | 0.626 |  |
|  |  | **Off-label reduced dose** | 0.9 | 1.54 (0.27 – 8.63) |  |  |
|  | **Secondary outcome** | | | | | |
|  | **Death from any cause** | | | | | |
|  | **<2** | **On-label standard dose** | 0.7 | 1 (references) | 0.371 | 0.348 |
|  |  | **Off-label reduced dose** | 1.7 | 1.92 (0.45-8.05) |  |  |
|  | **≥2** | **On-label standard dose** | 0.4 | 1 (references) | 0.240 |  |
|  |  | **Off-label reduced dose** | 1.9 | 2.67 (0.51-13.82) |  |  |
| **Safety** | **Primary outcome: Major bleeding** | | | | | |
|  | **<2** | **On-label standard dose** | 0.4 | 1 (references) | 0.947 | 0.883 |
|  |  | **Off-label reduced dose** | 0.7 | 1.07 (0.11-10.0) |  |  |
|  | **≥2** | **On-label standard dose** | 0.6 | 1 (references) | 0.592 |  |
|  |  | **Off-label reduced dose** | 1.3 | 1.53 (0.32-7.35) |  |  |
| **CKD-EPI eGFR** | | | | | | |
| **Effectiveness** | **Primary outcome: Stroke/systemic embolism** | | | | | |
|  | **≥60 mL/min/1.73m^2^** | **On-label standard dose** | 1.0 | 1 (references) | 0.060 | 0.418 |
|  |  | **Off-label reduced dose** | 0.2 | 0.11 (0.01 – 1.09) |  |  |
|  | **<60 mL/min/1.73m^2^** | **On-label standard dose** | 0.5 | 1 (references) | 0.323 |  |
|  |  | **Off-label reduced dose** | 1.8 | 2.96 (0.34 – 25.49) |  |  |
|  | **Secondary outcome** | | | | | |
|  | **Death from any cause** | | | | | |
|  | **≥60 mL/min/1.73m^2^** | **On-label standard dose** | 0.5 | 1 (references) | 0.322 | 0.881 |
|  |  | **Off-label reduced dose** | 1.8 | 1.86 (0.54-6.37) |  |  |
|  | **<60 mL/min/1.73m^2^** | **On-label standard dose** | 0.5 | 1 (references) | 0.330 |  |
|  |  | **Off-label reduced dose** | 1.8 | 3.06 (0.32-29.13) |  |  |
| **Safety** | **Primary outcome: Major bleeding** | | | | | |
|  | **≥60 mL/min/1.73m^2^** | **On-label standard dose** | 0.6 | 1 (references) | 0.918 | 0.579 |
|  |  | **Off-label reduced dose** | 1.0 | 0.92 (0.19-4.37) |  |  |
|  | **<60 mL/min/1.73m^2^** | **On-label standard dose** | 0.5 | 1 (references) | 0.518 |  |
|  |  | **Off-label reduced dose** | 1.2 | 2.16 (0.20-22.69) |  |  |

*Adjusted for age, sex, hypertension, diabetes mellitus, heart failure, CKD, prior stroke or TIA, prior bleeding, malignancy, antiplatelet use, anemia

Abbreviation: AF, atrial fibrillation; CI, confidence interval; HR, hazard ratio; IR, incidence rate; MI, myocardial infarction; PY, person-years; TIA, transient ischemic attack.

**Figure S1. Standardized mean differences before and after inverse probability of treatment weighting**


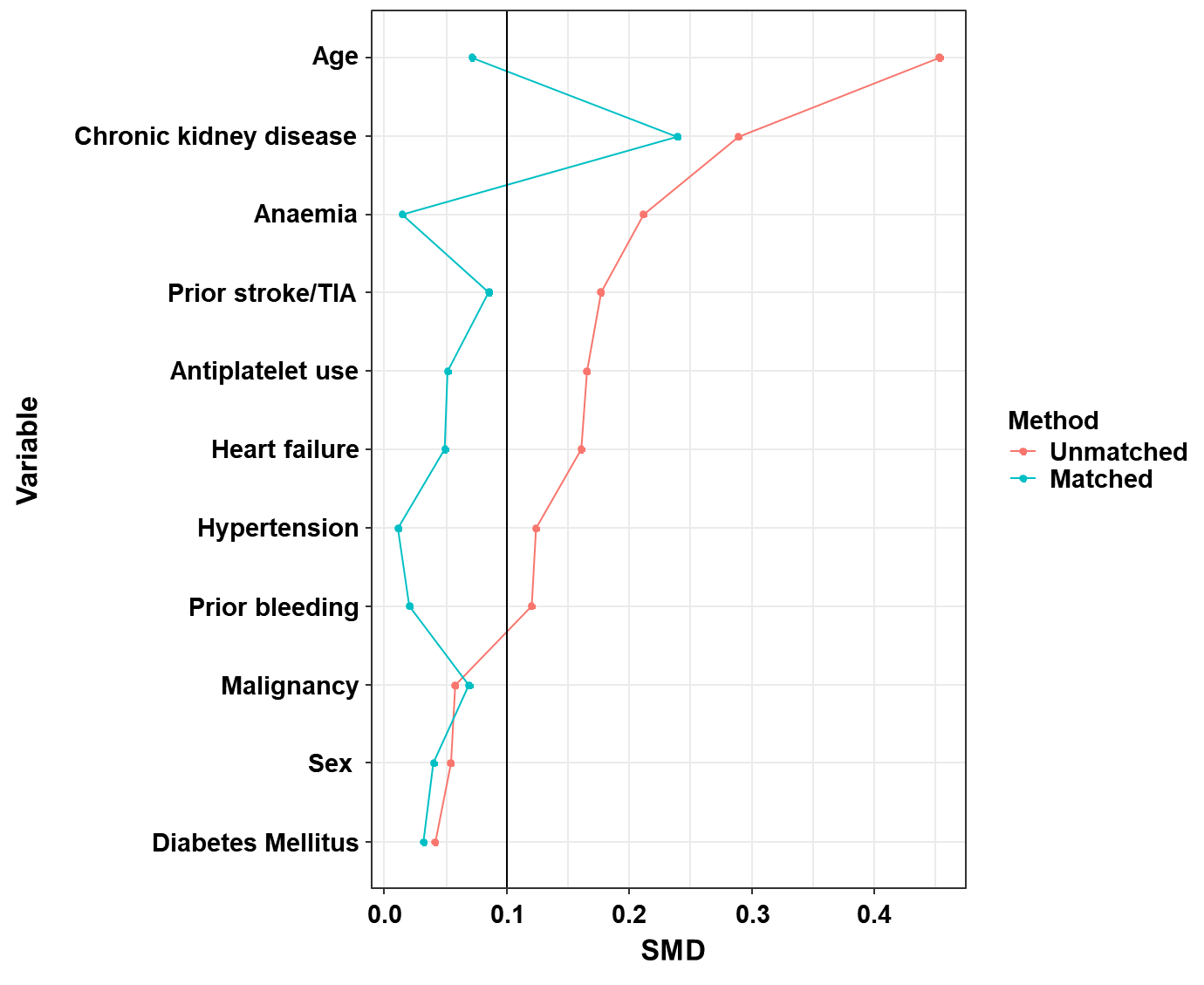


Abbreviation: SMD, standardized mean difference; TIA, transient ischemic attack.

**Supplemental References**

1. Choi J, Lee SR, Kwon S, Ahn HJ, Lee KY, Park JS, et al. Clinical characteristics of apixaban prescription in AF patients with single dose-reduction criterion: the ASPIRE (efficAcy and safety of aPixaban in rEal-world practice in Korean frail patients with atrial fibrillation) study. Front Cardiovasc Med. 2024;11:1367623.

2. Granger CB, Alexander JH, McMurray JJ, Lopes RD, Hylek EM, Hanna M, et al. Apixaban versus warfarin in patients with atrial fibrillation. N Engl J Med. 2011;365(11):981-92.

3. Lane DA, Lip GY. Use of the CHA(2)DS(2)-VASc and HAS-BLED scores to aid decision making for thromboprophylaxis in nonvalvular atrial fibrillation. Circulation. 2012;126(7):860-5.

4. Schulman S, Kearon C. Definition of major bleeding in clinical investigations of antihemostatic medicinal products in non-surgical patients. J Thromb Haemost. 2005;3(4):692-4.

5. Heinze G, Jüni P. An overview of the objectives of and the approaches to propensity score analyses. Eur Heart J. 2011;32(14):1704-8.

6. Austin PC, Stuart EA. Moving towards best practice when using inverse probability of treatment weighting (IPTW) using the propensity score to estimate causal treatment effects in observational studies. Stat Med. 2015;34(28):3661-79.

7. Zhang Z, Kim HJ, Lonjon G, Zhu Y. Balance diagnostics after propensity score matching. Ann Transl Med. 2019;7(1):16.
